# Coaxial Monitoring of AISI 316L Thin Walls Fabricated by Direct Metal Laser Deposition

**DOI:** 10.3390/ma14030673

**Published:** 2021-02-01

**Authors:** Vito Errico, Sabina Luisa Campanelli, Andrea Angelastro, Michele Dassisti, Marco Mazzarisi, Cesare Bonserio

**Affiliations:** Department of Mechanics, Mathematics and Management, Politecnico di Bari, Via Orabona 4, 70125 Bari, Italy; sabinaluisa.campanelli@poliba.it (S.L.C.); andrea.angelastro@poliba.it (A.A.); michele.dassisti@poliba.it (M.D.); marco.mazzarisi@poliba.it (M.M.); cesare.bonserio@poliba.it (C.B.)

**Keywords:** direct metal laser deposition, melt pool, process monitoring, image processing

## Abstract

Direct metal laser deposition (DMLD) is an additive manufacturing technique suitable for coating and repair, which has been gaining a growing interest in 3D manufacturing applications in recent years. However, its diffusion in the manufacturing industry is still limited due to technical challenges to be solved—both the sub-optimal quality of the final parts and the low repeatability of the process make the DMLD inadequate for high-value applications requiring high-performance standards. Thus, real-time monitoring and process control are indispensable requirements for improving the DMLD process. The aim of this study was the optimization of deposition strategies for the fabrication of thin walls in AISI 316L stainless steel. For this purpose, a coaxial monitoring system and image processing algorithms were employed to study the melt pool geometry. The comparison tests carried out highlighted how the region-based active contour algorithm used for image processing is more efficient and stable than others covered in the literature. The results allowed the identification of the best deposition strategy. Therefore, it is shown how this monitoring methodology proved to be suitable for designing and implementing the right building strategy for DMLD manufactured 3D components. A fast and stable image processing method was achieved, which can be considered for future closed-loop monitoring in real-time applications.

## 1. Introduction

The direct metal Laser deposition (DMLD) is an additive manufacturing process based on laser cladding, which mainly focuses on 3D manufacturing and repair applications. DMLD has a high potential for component production through 3D deposition strategies for the creation of clads with quite good microstructures less prone to cracks and also to build non-homogenous structures [1].

DMLD uses a laser beam to create a melt pool on a metal substrate in which a metal powder or a metal wire is added. The generation of an appropriate melt pool during the process is essential to achieve a strong bond between the cladding material and the substrate. The physical mechanisms behind the DMLD process are the creation of a high-temperature spot during the process inducing the melting of the substrate and the fed material, both contributing to the biphasic (liquid + solid regions) melt pool growth [2,3,4]; when the laser beam moves away, the heat dissipation into the substrate causes rapid cooling of the trailing molten material (leaving a semi-circular trace of solidified material) and the advancement of the leading solidification front [5].

However, from an economic point of view, the spreading of the process in the industrial sector is slowed down by technical challenges still to be solved. The main issues are the sub-optimal quality (in terms of dimensional accuracy and surface regularity) of the final parts and the low repeatability, which make this process inadequate for high-value applications requiring high-performance standards.

In order to overcome these problems and improve the product quality and the process repeatability, several research groups investigated in-situ process monitoring, characterization, and real-time process control. Process monitoring involves observation, information gathering, understanding of physical phenomena occurring in laser–material interactions, and finally, the development of automated process control systems. This is also an essential method to reduce costs and the amount of material waste. A first classification distinguishes between near-IR thermal monitoring (using thermal imaging cameras, pyrometers, and bolometers) and optical monitoring by image analysis (using camera with active-pixel sensors, such as CMOS or CCD sensors, or photodiode sensors) of the melt pool. Monitoring methods can be also classified, by regarding the topological setup, as on-axis (coaxial) and off-axis with respect to the laser beam. Equipment with different technical characteristics can detect different aspects of the process, and by combining different systems, more sophisticated analyses can be carried out [6].

Although several papers in the literature focus on monitoring the melt pool, few of them adopt the algorithm proposed in this study (named active contour) to retrieve the melt pool size in the DMLD process. This algorithm consists of iterative modifications of an initial contour until reaching the minimization of an energy functional. The active contours logic was widely explored and applied in numerous applications, such as image segmentation, visual tracking, etc. It generally leads to better results than classical image segmentation methods [7]. Nevertheless, a challenge for this methodology regards the sensitivity of the geometrical results to the contour initialization [8]. A methodology evolution, employable to improve the algorithm performances, is the region-based active contours, which models different zones in terms of intensity and sets the region boundaries as the transition between different zones. The algorithm aims to identify each region of interest applying a region descriptor leading the evolution of the active contour.

The aim of this study was the optimization of deposition strategies for the fabrication of thin walls in AISI 316L stainless steel. For this purpose, a coaxial monitoring system and image processing algorithms were employed to study the melt pool geometry. The comparison tests carried out highlighted how the region-based active contour algorithm, used for image processing, is more efficient and stable than others covered in the literature. The algorithm was also compared with three different image segmentation techniques analyzed in the literature—threshold segmentation, Canny edge, and edge-based active contour. It was shown how the region-based active contour outperforms the other in terms of processing speed, resolution, and edge detection accuracy. This algorithm was previously employed by Lei et al. [9] to investigate the melt pool geometry in high-power diode laser deposition with a rectangular laser spot, using an off-axis vision system. A similar methodology was employed in the present study but, despite previous studies, a coaxial CCD camera system integrated into the deposition head was used. It was shown how coaxial monitoring avoids the pre-setting phase for perspective correction of the captured images. However, this methodology is particularly suitable for the DMLD process monitoring as it is less affected by the field of view occlusions. The results allowed the identification of the best deposition strategy.

Moreover, a short review of the thermal and optical techniques used for in-situ monitoring of the melt pool during the DMLD process is presented, in order to highlight recent researches on existing sensing systems and on analysis of product quality.

## 2. Review of Main In-Situ Monitoring Systems

### 2.1. In-Situ Thermal Monitoring

Through thermal analysis, it is possible to identify defects such as porosity, lack of fusion, or surface irregularities, coming from non-optimal heat dissipation conditions. In current practice concerning thermal monitoring, a series of studies have been carried out. R.D. Murphy and E.C. Forrest [10] reviewed in-situ temperature measurement techniques for additive manufacturing technologies. In their study, the critical points of thermal monitoring were highlighted. Practically, the temperature field of a body is not easy to detect, due to the variable emissivity during laser interaction, with consequences on measurement accuracy. Owing to the complexity of emissivity definition, monochromatic pyrometers are not recommended for applications where accurate measurements are required. However, some studies in the literature using monochromatic pyrometers are reported. Emamian et al. [11] performed thermal monitoring of the microstructure and carbide morphology of Fe–Ti–C metal matrix composites, establishing in their experiments a constant emissivity without needing to know the correct temperature. The authors employed the monochromatic pyrometer for a comparative study on cooling rates and temperatures of the melt pool under different conditions, setting the emissivity to a known value. An alternative way involves the pyrometer calibration through a black body, as in the case of Smurov et al. [12]. A high-temperature black body calibration source (MIKRON M390, Boudry, Switzerland), with an emissivity of 1 in the band between 0.65 µm to 1.8 µm is used. Because the emissivity of the body was known, the measured temperature made it possible to calibrate with respect to the pyrometer. Usually, for DMLD processes, “ratio pyrometers” are used to measure the energy of the infrared radiation emitted by the body at different wavelengths. Measurements made by such type of pyrometers are sensitive to measurement noise. For this reason, it is recommended to use dual-color pyrometers, which are the most widespread for industrial applications [13]. Song et al. [14] stated that by selecting wavelengths of 1.3 µm and 1.64 µm, an accuracy of ±10 °C is achieved at temperatures between 1000 °C and 3000 °C. Shuang Liu et al. [15] also conducted monitoring of high-power diode laser cladding using a pyrometer and an infrared camera in order to visualize the interaction of the laser beam with the powder flow and to record the temperature of the melt pool. In this study, the influences of the main process parameters on the thermal behavior of the melt pool were investigated. It turned out that the trail of the melt pool temperature was increased by a rise in laser power or a decrease in carrier gas flow rate.

### 2.2. In-Situ Optical Monitoring

As mentioned, another type of monitoring is carried out via optical techniques, which are used mainly to detect the geometry of the melt pool. Analyzing the literature, it was found that optical systems and methods are used more than thermal methods for many advantages—the possibility of real-time and multi-functional acquisitions (i.e., temperature and images of the melt pool), intuitiveness, and flexibility [16]. The melt pool can be observed by digital cameras, especially CMOS, CCD, and IR, which allow a visual study of the target. From the acquired images, the geometrical features of the melt pool can be measured after a post-processing phase. During the process, an attempt to keep primary dimensions as constant as possible is made in order to have a stable deposition. However, as in the case of temperature measurements, one of the main challenges for these cameras is the calibration of the sensors and threshold values in image processing. With infrared cameras, images are captured in grayscale, based on brightness intensity. Each pixel has its grey value, and to calculate the width and length of the melt pool, it is necessary to identify the value corresponding to the edge of the melt pool. Lei et al. [9] proposed the analysis and modeling of the melt pool for high-power diode laser cladding with a rectangular beam spot. The effects of the process parameters on the final geometrical characteristics were investigated. A camera with a frame rate of 31 frames/s was employed to monitor the melt pool during the process. The region-based active contours method, which was able to isolate regions characterized by nearly homogeneous intensities, was used to detect the melt pool boundaries. Results showed that the melt pool size (i.e., width and depth) increases with increasing laser power while increasing translation speed leads to a decreasing in the melt pool size. Hassler et al. [17] used a thresholding technique based on the thermal behavior of the melt pool. They calibrated an infrared camera using the emissivity trend of a black body and extracted the edge of the melt pool by defining the temperature of the solidus–liquidus boundary. Other methods reported in the literature are related to “trial and error” image processing methods. Akbari et al. [18] set the grayscale threshold value by comparing the nominal width of deposited tracks with the width measured in the images, reporting a value of 80. The authors observed some glares upon the melt pool caused by hot incoming powder particles. Since these glares could generate measurement errors, a low-pass filter is applied to the images using the fast Fourier transform (FFT) technique. Regular intensity variations in the image corresponded to low frequencies, while abrupt and rapid intensity variations, such as those induced by glaring or noisy pixels at the edge of the melt pool, corresponded to high frequencies. A low-pass cut-off frequency of 5% was used to remove the noise. Finally, the edge of the melt pool was extracted, and all possible circumferences approximating the molten pool were detected in order to measure the largest section. The width of the melt pool was estimated as the largest of the calculated diameters. Ocylok et al. [19] also surveyed the correlation between the geometry of the melt pool and the main process parameters of the laser metal deposition process by coaxial process monitoring. A CMOS camera was used to record back reflections of the melt pool, which was useful for further analyses. It was pointed out that thresholding has a great influence on the result of image binarization, and when using a low threshold value, an over-estimation of the melt pool size was obtained. The same threshold value was applied for all the analyzed images and results found a constant deviation of 150 µm between the width of the deposited track and the width measured in the images. Furthermore, the widening of the track is caused by heat accumulation and viscosity reduction of the melt pool and by the presence of sparks. The effects of glares on width and length measurement of the melt pool were reported to be less than 0.5%. In this study, the results also showed that the laser power has a positive correlation with the melt pool size, while the correlation between translation speed and melt pool size was negative. Increasing the powder mass flow increases the thickness of the single track almost linearly and the penetration depth decreases, leading also to a small reduction in the melt pool size. The effect of preheating of the substrate (up to 300 °C) was also evaluated, proving that an increase of the melt pool size by more than 20% at all examined laser power values was observed. Sampson et al. [20] implemented a new image processing algorithm for improving the accuracy and performance of melt pool measurements. It was based not on emissivity or material dependency but on a parametric study, comparing it to a study based on emissivity. The new algorithm uses the phenomenon of directional emittance to calculate the width of the melt pool. For monitoring it, a NIR CMOS vision camera with a 135 nm UV/VIS cut-off imaging filter was installed coaxially to the laser beam on the deposition head. The results highlighted that the melt pool edge often occurs at different thresholds. For this reason, this new technique detected the edge of the melt pool without problems, compared to conventional emissivity-based techniques. Vandone et al. [21] split the process of image analysis into two parts. The images were first corrected because the brightness was elevated due to rising vapors. These vapors trapped the radiation from the substrate, generating a light halo that deceived the measurements. The pixel intensity distribution was analyzed, and the skewness index was evaluated. Negative indices indicated that the image was composed of bright pixels and revealed a deceptive intensity. In order to solve the problem at this early stage of the process, a corrective factor was applied to the images. In a second step, the images were analyzed to extract the geometry of the melt pool. For each image, the threshold value was calculated using the Otsu method. To extract spark data, the local thresholding approach was followed. Sparks were excluded based on the fact that they were composed of pixels with extremely variable intensity. The same authors, in another study [22], carried out coaxial monitoring of the melt pool by using a FLIR Grasshopper 3 (Wilsonville, OR, USA) camera. They performed V-track depositions tests to demonstrate that the image intensity signal was strongly linked to the local increase in power density, which occurred when the deposition head decelerated to change direction. This phenomenon generated an over deposition. Therefore, an image processing algorithm and different setup solutions to optimize image signals were studied to detect this phenomenon in real-time and generate a feedback signal to adjust the process parameters. Results showed that a narrow band filter in addition to the image-processing algorithm and an optimal camera exposure time could improve the detection of errors during the process. Ding et al. [23] conducted experiments using two different cameras—CCD and IR. An IR filter (>650 nm) was applied to the CCD camera to eliminate disturbances in the acquisitions due to the presence of powder above the melt pool. They calibrated the CCD camera by comparing the acquisitions of the IR camera. Both the CCD and infrared cameras were used to acquire a video from the molten pool in absence of powder and under the same process conditions. Because the edge of the melt pool was determined by the melting temperature of the material, the emissivity was constant along the whole contour and the edge temperature was described by an isotherm. In order to determine this isotherm, the images extracted from the videos of the two different cameras were overlapped. A video was acquired by replacing the IR filter with a 532 nm bandpass filter and illuminating the melt pool with a 5 W green laser. By overlapping the greyscale images on the IR image, the greyscale threshold value for IR images was established to 97. This value was verified by experiments carried out at different scanning speeds without powder and the results corresponded to the size of the deposited tracks with a deviation of ±0.1 mm. Moreover, in the research of Kledwig et al. [24], the intensity distribution of the signal coming from the melt pool surface was considered for the monitoring of the directed energy deposition process. The intensity distribution was monitored using a coaxial CCD camera. The melt pool area was estimated taking into account the number of pixels (NOP) having intensities larger than a predefined threshold. This study showed how the minimum specific energy needed for a stable process can be determined. Outcomes indicated that a NOP having an intensity larger than a threshold intensity was a sign of an unstable cladding process. This result has been attributed to the variation of the working distance and this could be used as a warning signal for the automatic stop of processing.

As concerning off-axis monitoring systems, recently Garmendia et al. [25] proposed a closed-loop control system that allowed the adjustment of the height of the deposited layer and recalculate the deposition paths during repositioning. The monitoring was performed using a structured light scanner. The used device was an HP SLS3 (Palo Alto, CA, USA) (with an accuracy of 0.05 mm). The monitoring process implemented in this work was designed on the interruption of printing, after a predetermined number of layers, and on the consequent scanning of the previously made depositions. Once scanned, it compared the images to the related CAD profiles. Then, if necessary, a new toolpath was generated by updating the layer height to adapt the new deposition to the part growth. A new coordinate system was updated for each scan. The results showed that the usage of different coordinate systems for each scan improved the overall accuracy. This system has generated parts with better dimensional features. Hsu et al. [26] proposed an inspection system to measure the height of the clad based on three digital cameras. These were placed at the same distance from each other and about 150 cm from the center of the platform and inclined about 15 degrees from the vertical. In this system, a calibration bar was used to rectify the field of view and perspective effects of the trinocular system. An image processing technique was previously used to isolate the nozzle and the melt pool. Findings showed how the clad height was estimated based on the distance between two reference points located at the nozzle tip and on the centroid of the melt pool. The accuracy of the system was compared with 3D scan models (GOM ATOS-Compact scan, Braunschweig, Germany), giving an error of 4.2%.

## 3. Materials and Methods

### 3.1. Experimental Setup

**Figure 1:**
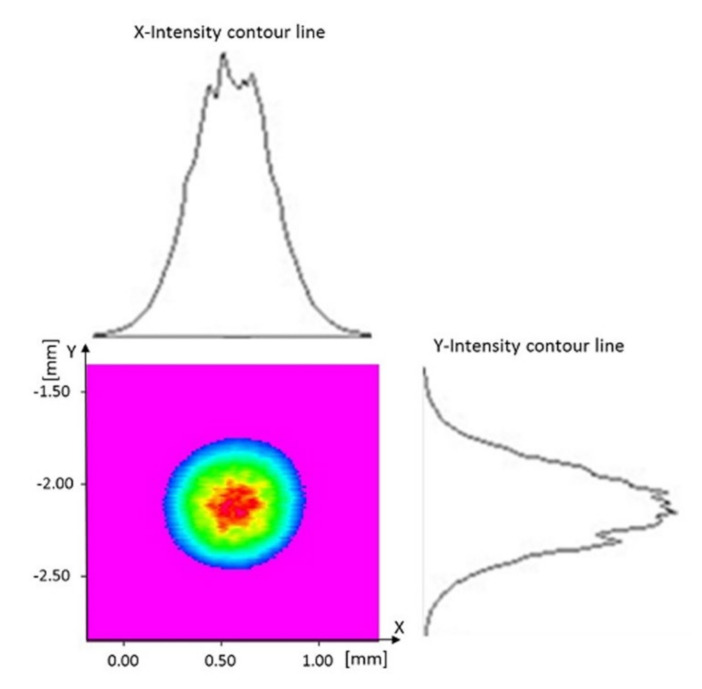
Intensity distribution profiles for the laser beam, after passage through the fiber, as a function of radial coordinates and cross-sectional plot.

In this study, thin-wall depositions with different depositions strategies were built. The substrate was an AISI 304 stainless steel plate, whose chemical composition is shown in Table 1, with a size of 100 mm in length, 80 mm in width, and 2 mm in thickness. The filler metal was an AISI 316L stainless steel powder, whose composition is shown in Table 2, presenting spherical particles produced by gas atomization by LPW South Europe (Widnes, UK). The particle size distribution (PSD) of the powder, as certified by the manufacturer, is shown in Table 3. As shown in Table 3, the D value set at D10, D50, and D90 (10%, 50%, and 90%) indicates the diameter of the particles, where 10%, 50%, or 90% of the population lies below a certain size. The granulometry of the gas atomized powder is in the range of 15–45 μm. The experimental work was carried out by using a direct metal laser deposition prototype machine, which includes a 4-kW fiber laser source (*λ* = 1.070 μm, Ytterbium Laser System YLS by IPG Photonics, Oxford, MA, USA), a five-axis handling system, and a powder supply system by GTV (Luckenbach, Germany). The laser head was equipped with an optical collimator. The focal length of the collimation lens and the focusing lens were respectively 100 mm and 200 mm. The beam and intensity distribution profiles are shown in Figure 1. The laser beam was guided from the laser source to the deposition head through an optical fiber with a diameter of 100 μm. A coaxial Argon flow with a flow rate equal to 10 L/min was used to shield the working area. The powder was supplied by an external powder feeder that used argon as a carrier gas and was injected through a coaxial nozzle into the melt pool.

As mentioned above, thin-wall depositions were performed with the same process parameters that were obtained from prior studies and evaluated as appropriate for multilayer depositions. The process parameters that were kept constant based on previous tests [27] are listed in Table 4.

**Figure 2:**
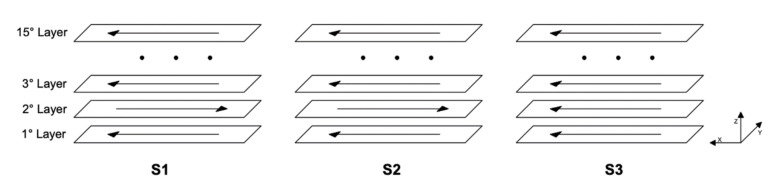
Schematic representation of three different deposition strategies.

The thin walls, consisting of 15 layers, were built with the following three different deposition strategies (see Figure 2):

S1: two-way without dwelling time;
S2: two-way with 10 s of dwelling time between two consequent paths;
S3: one-way with 12.5 s of dwelling time, including 2.5 s for the return to the beginning of each path.

The dwelling time is the waiting time between two consequent paths in which the laser is off. Two replications were performed for each deposition strategy.

A coaxial CCD camera (IDS UI-6230RE-M-GL PoE Rev.3, Obersulm, Germany) integrated into the deposition head, which allowed the laser beam path to be coaxially followed, was employed to monitor the melt pool during the fabrication of thin walls.

The camera was characterized by a mono CCD sensor, an acquisition frequency of 40 frames per second, and an optimal resolution to control the process of 1024 × 768 pixels. From the videos acquired by the camera, several series of frames were extracted and processed using algorithms implemented in the MATLAB software, for extracting the geometrical dimensions of the melt pool.

In order to investigate the effects of the three proposed deposition strategies on the final quality of the thin walls, a geometrical analysis on cross sections of the samples was carried out. These were obtained by cutting the samples in a transverse direction using the AbrasiMet 250 Buehler (Lake Bluff, IL, USA) metallographic cut-off machine. A polishing process was carried out to make the cross-section surface highly reflective and free of scratches and deformations. To characterize the samples in terms of microstructure, geometry, and defectiveness, they were etched by Glyceregia reagent (5 mL HNO_3_, 10 mL glycerol, and 15 mL HCl), which allowed the metallurgical structure to be observed. The samples prepared in this way were examined by a Nikon Eclipse MA200 inverted optical microscope (Nikon Corporation, Tokyo, Japan) for micrographic analysis.

### 3.2. Analysis and Characterization of the Melt Pool

For each deposition test, a video of the molten pool evolution was acquired using the coaxial CCD camera with an acquisition rate of 40 frames per second. The coaxial configuration is particularly suitable for the DMLD process monitoring because it is less affected by the field of view occlusions and perspective distortion [21]. Image sequences were extracted from the acquired videos and subsequently processed and analyzed using dedicated algorithms developed in a MATLAB environment to identify and isolate the melt pool. For each deposited layer, frames were extracted and evaluated at three different key points—in the beginning, in the middle, and at the end of the deposition path, 25%, 50%, and 75% of the total length of the single track, respectively. The melt pool shape monitoring was performed by extracting its geometrical characteristics using advanced image processing techniques.

In particular, the study focused on the identification of the melt pool area through an algorithm based on the active contour method. This technique, previously used by Lei et al. [9], consists of an iterative method starting with the definition of the zero-level contour in the form of a closed curve known as a mask. Subsequently, the zero-level contour iteratively evolves and adapts by applying shrinking/expanding operations called “contour evolution” driven by the minimization of an energy function [28].

**Figure 3:**
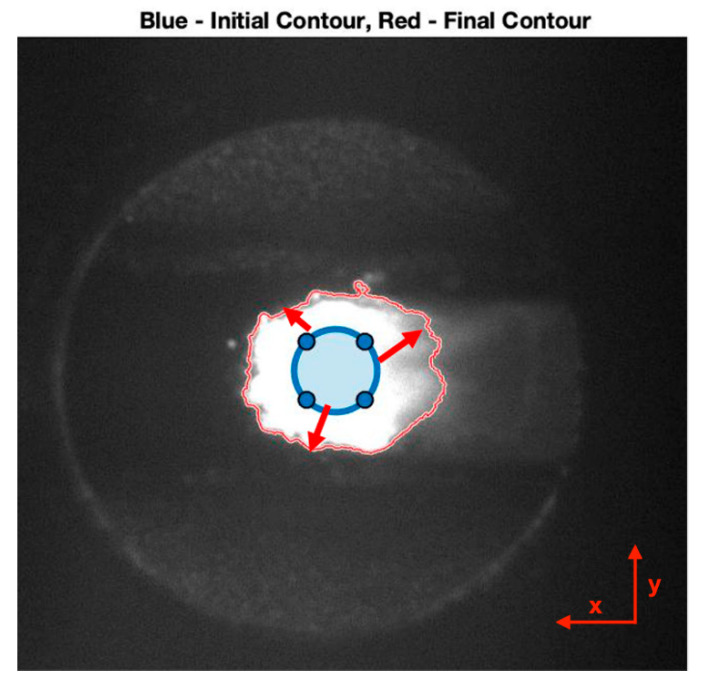
Automated detection of a melt pool edge from a video frame using the active contour algorithm.

In this study, a region-based algorithm was implemented, which aims to identify each region of interest using a region descriptor that guides the evolution of the active contour. The algorithm works by segmenting the image in order to connect regions with homogeneous properties. Among the various selectable shapes for the zero-level contour (elliptical, circular, rectangular, freehand), the circular shape was chosen because it is similar to the incident laser spot. Figure 3 shows an example of an active contour where both the starting contour (blue) and the extracted melt pool contour (red) are represented. The brightness gradient at the top of the molten pool is steep due to intense heat dissipation through the substrate. The high brightness regions on both sides of the melt pool could be a consequence of the high emissivity, proper of the non-molten particles, and oxides [9,10,11,12,13,14,15,16]. Similar considerations were made by Doubenskaia et al. [29], who considered the high thermal emission in the peripheral area of the melt pool due to oxides and other non-metallic inclusions that are usually concentrated in that region. The weak brightness gradient detected in the trailing part of the melt pool is describing lower and quite uniform temperatures owing to cooling and solidification of the material in that area.

## 4. Results and Discussion

### 4.1. Comparison of Image Segmentation Techniques

**Figure 4:**
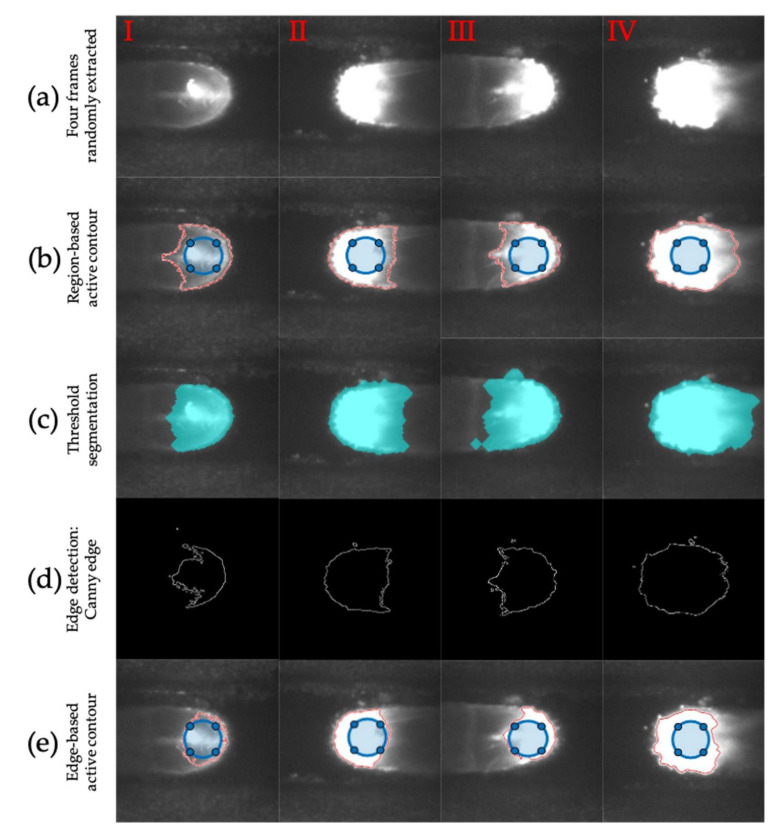
Comparison of the region-based active contour with other techniques: (**a**) four frames randomly extracted from the whole deposition; (**b**) region-based active contour; (**c**) threshold segmentation; (**d**) Canny edge; and (**e**) edge-based active contour.

In order to illustrate the advantages of the proposed image analysis algorithm, the region-based active contour was compared with three image segmentation techniques analyzed in the literature—the threshold segmentation, the Canny edge, and the edge-based active contour. Figure 4 shows the results of the algorithms comparison and the different melt pools identified. Four frames randomly extracted from the complete deposition were analyzed (see Figure 4a). As shown in Figure 4b–d, the region-based active contour algorithm, compared to threshold segmentation and Canny edge, demonstrated a superior accuracy in terms of edge detection and resolution. These results stem from the following advantages of the region-based active contour over other algorithms [7]:

- achievement of sub-pixel accuracy for detected object boundaries;
- incorporation of prior image knowledge, such as intensity distribution (useful for robust image segmentation);
- realization of smooth and closed contours as segmentation results, which are crucial and easily manageable for further applications such as shape analysis and feature recognition.

The threshold value was chosen to be 80, as mentioned in the study by Akbari and Kovacevic [18]. However, the classical image segmentation methods (edge detection and thresholding) present problems due to the incapability to approximate edges with a single binary threshold, as mentioned in [7,20]. The latter operates by finding discontinuities in the brightness intensity of the image. These algorithms detect intensity discontinuities and identify the edge between two regions characterized by different properties (e.g., the intensity of pixels) as the limit. Therefore, because these techniques are based on a locally derived analysis, they are not very effective in presence of weak object boundaries or noisy patterns, as the images examined in this study [7,30].

In fact, independently of the selected threshold value, a part of the surface of the substrate is erroneously identified as a melt pool part and vice versa, as shown in Figure 4c,d. The last drawback also appeared in the edge-based active contour algorithm (see Figure 4e), which uses the local edge information to attract the active contour to the edge to be detected; it is not very effective in the presence of objects with heterogeneous feature profiles or images with non-homogeneous intensity, as in the images obtained in our study [7,9]. Hence, among the four techniques, the region-based active contour method gave a higher performance in terms of resolution, edge detection accuracy, etc.

In addition, a comparison of processing performances in terms of image segmentation computing time was performed. The processing times of different algorithms were evaluated (see Table 5). For each image, the processing time of the region-based active contour method was the shortest one, as shown in Table 5. The edge-based method is not supported for color or multi-channel images and therefore requires image pre-processing, thus increasing the processing time. Finally, in order to evaluate the outcome accuracy of proposed algorithms, four levels of image processing quality were defined in Table 5—very good, quite good, acceptable, and bad.

In summary, the analysis indicates that the region-based active contour method outperforms the other three techniques in terms of processing speed, resolution, and edge detection accuracy.

### 4.2. Effects of Deposition Strategies on Melt Pool Geometry

**Figure 5:**
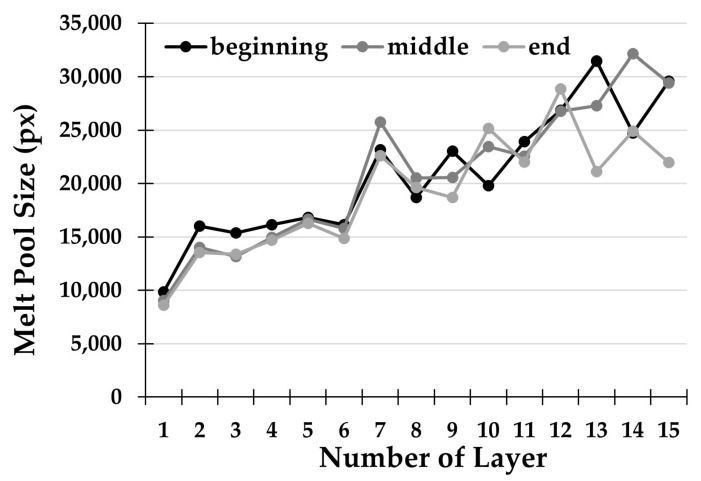
Melt pool size for each deposited layer with strategy S1.

The melt pool size is an important feature for the characterization of DMLD depositions. Figure 5 shows the effect of the two-way deposition strategy without dwelling time (S1) on the final melt pool area. Each plotted value is the average of the measurements taken on the two replications performed with the same deposition strategy.

In order to investigate the effects of the above-mentioned deposition strategy on the melt pool size of the thin wall built, geometrical analysis of the melt pool was carried out. In particular, three distinctive regions (beginning, middle, and end) were observed along every single track, specifically at a distance of 25%, 50%, and 75% of the total length of the single track. Results show an increasing trend in the size of the melt pool with increasing deposited layers. As stated by Yang et al. [31], two-way laser scanning generates an excessive heat accumulation in a specific area of the workpiece in the proximity of the reversal point for scanning direction. This causes a rise in the substrate temperature and therefore the size of the melt pool is rapidly increased. In addition, there is no dwelling time in the deposition under examination, thus amplifying this effect. The analysis of the three key points considered for each deposited layer revealed the variation in the molten pool size along with the single deposition. In strategy S1, there is a substantial coherence of the dimension in the first layers. On the other hand, the variation in size becomes extremely marked, exceeding 10,000 px in the last tracks. This effect is due to the uneven accumulation of heat inside the workpiece.

**Figure 6:**
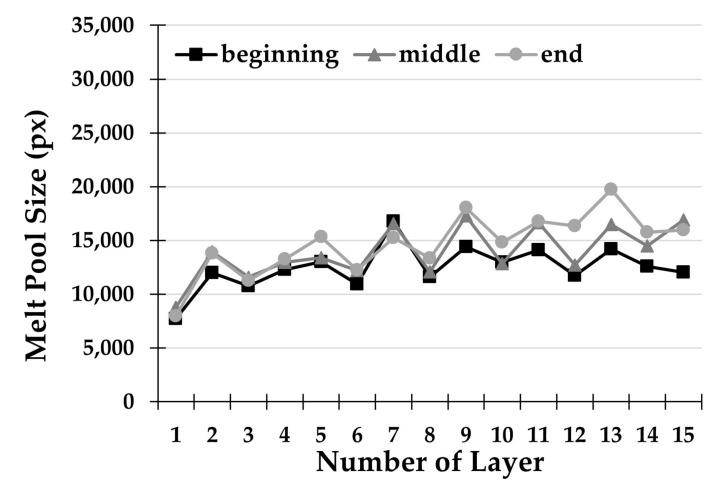
Melt pool size for each deposited layer with strategy S2.

Figure 6 shows the trend of the extracted and measured melt pool areas in a two-way deposition strategy with 10 s of dwelling time (S2). Results show that even for two-way deposition with dwelling time there is an increasing trend in the melt pool area as the deposited layers increase. However, in this case, the increasing trend is less marked than the previous one, because when the laser is switched off, there is enough waiting time between depositions for heat sinking. In this way, initial thermal conditions are nearly restored and kept as constant and repeatable as possible throughout the process.

The robustness analysis of the DMLD process, performed by comparing the size of the three key points of each track, reveals significantly more consistent values for the S2 strategy. In fact, there is a maximum deviation of the molten area of 5520 px in layer 13. Therefore, the strategy with waiting times is much more stable in the construction of components consisting of several layers, because it aims at diminishing the detrimental heat accumulation effects in thin walls.

**Figure 7:**
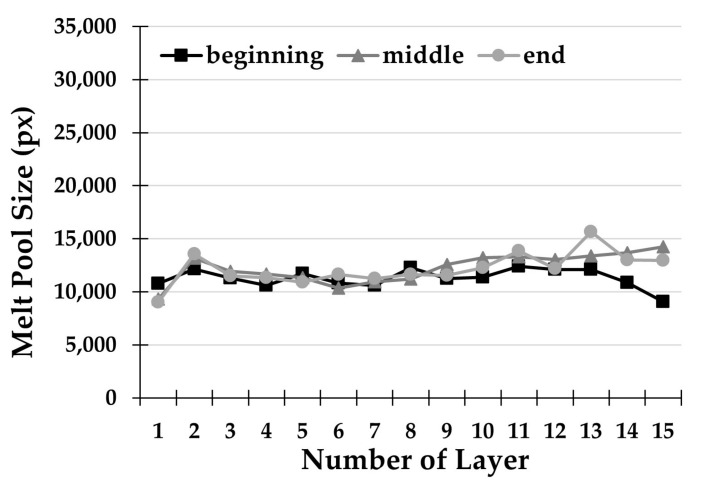
Melt pool size for each deposited layer with strategy S3.

Finally, Figure 7 shows the trend of the extracted and measured melt pool area, in the one-way deposition strategy with 12.5 s of dwelling time (S3). In this case, the trend of the melt pool size is approximately constant with the deposited layers. This tendency is due to the combination of waiting times and constant deposition direction that are carried out with this specific deposition strategy. The unidirectional strategy avoids the formation of heat accumulation points at the extremes of the thin wall due to the reversal of direction, where the end of a track is the beginning of the subsequent. A consequent more uniform heating of the part is obtained, practically consisting in a superposition of the same thermal field over time. This, combined with the previously defined effects of the waiting time, allows an effective diffusion of the accumulated heat.

As can be seen in Figure 7, the S3 strategy turns out to be extremely robust and coherent than the previous ones. There is a maximum variation of the melt pool area within the single layer in the order of 5090 px, and an average variation of the area along with all 15 layers of only 1725 px. This value is definitely lower than the previous ones (3542 px for S1 and 2543 px for S2), which makes the unidirectional strategy with dwelling times the most suitable and stable for the long processing.

**Figure 8:**
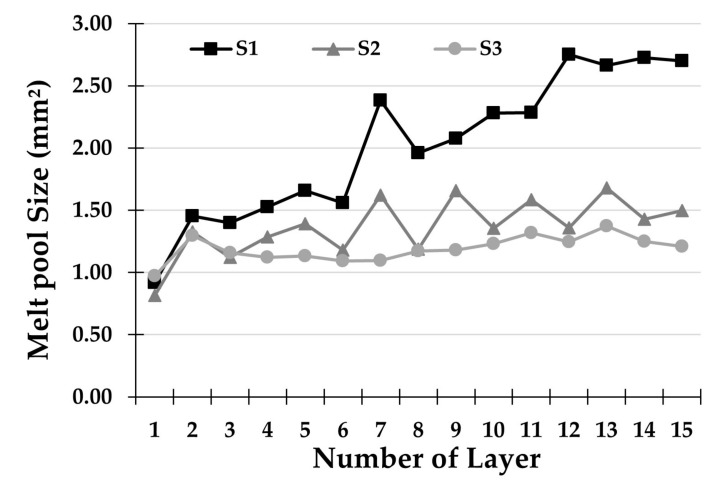
Comparison of melt pool sizes for each deposited layer for the three different strategies.

Moreover, by overlapping the results obtained by the three analyzed strategies (see Figure 8), it is clear that the dwelling time between consecutive layers is a key variable in the deposition process. In the graph, the areas of the melt pool have been converted from pixels to square millimeters, with a surface conversion: 10 px × 10 px = 1 mm^2^. This relationship was found through a calibration process, using a millimeter-sized sample. The individual trends in Figure 8 were obtained with the average areas for each layer deposited. The average areas for the three deposition strategies were compared and it was found that the smallest melt pool area is always recorded at the first layer of each strategy, while the largest area was found in the last layers of each treatment. The melt pool size seems to stabilize as the number of layers increases and this fact is more evident if the adopted strategy does not favor heat accumulation. More in detail, the strategy S1 increased the average melt pool area from 0.917 mm^2^ to 2.698 mm^2^, with a variation of 1.781 mm^2^. For the strategy S2, an increase in the average melt pool area was recorded, from 0.815 mm^2^ to 1.497 mm^2^, with a difference of 0.682 mm^2^. Finally, for the strategy S3, the melt pool area varied from 0.97 mm^2^ to 1.208 mm^2^ from the first to the last layer, recording the smallest increase of 0.238 mm^2^.

### 4.3. Analysis of Macrography Cross Sections

**Figure 9:**
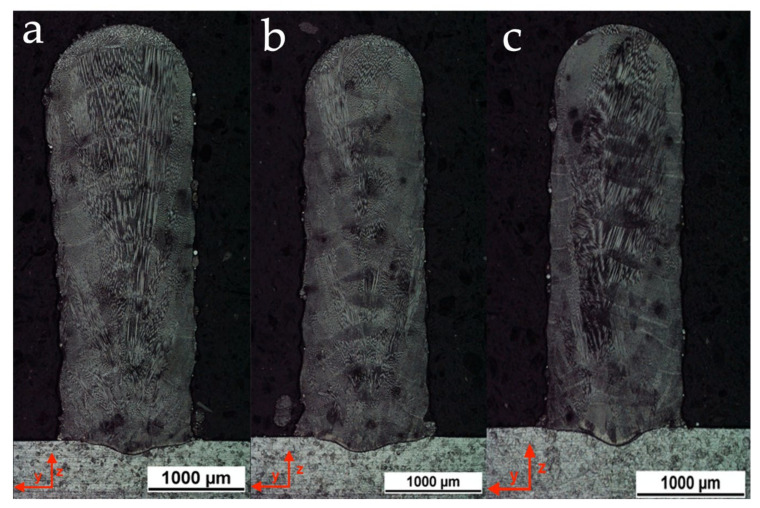
Macrographic cross section of the thin wall obtained using (**a**) strategy S1, (**b**) strategy S2, and (**c**) strategy S3.

In order to validate the image segmentation outcomes, a comparison with the cross-section macrographs of thin walls, which was realized using the above-mentioned strategies, was carried out. Figure 9a shows the cross section of the thin wall obtained using the deposition strategy S1. This macrography revealed that the width of the deposited thin wall increased as the deposited layers increased, confirming the trend determined in the melt pool assessment. On the other hand, Figure 9b shows the cross section of the thin wall obtained using the deposition strategy S2. This macrography also revealed that the width of the deposited thin wall increased as the deposited layers increased. However, the increase in width is much less pronounced than the previous. Finally, Figure 9c shows the cross section of the thin wall obtained with the strategy S3. This macrography once again confirms the outcomes of the melt pool monitoring, since the width of the deposited thin wall remains essentially constant along with the whole component.

## 5. Conclusions

In this work, coaxial monitoring of AISI 316L thin walls fabricated by direct metal laser deposition using different deposition strategies was carried out. A DMLD prototype machine with a coaxial CCD camera, integrated into the deposition head, was used for the geometrical characterization of the melt pool. A region-based active contour algorithm was implemented for image processing in order to analyze captured images and detect the melt pool area. The effects of the deposition strategies variations on melt pool features were analyzed. The following conclusions were drawn:

- The region-based active contour was compared with three image segmentation techniques analyzed in the literature—threshold segmentation, Canny edge, and edge-based active contour. Results show that the region-based active contour outperforms other algorithms in terms of processing speed, resolution, and edge detection accuracy;
- For the two-way deposition strategy without dwelling time (S1), as the deposited layers increased, a marked increase in the melt pool area was observed. The reason for achieving these results is the effect of bi-directional laser scanning, which generates excessive heat accumulation in the workpiece. In addition, there are no waiting times in the deposition under examination, so this effect was amplified.
- Concerning the two-way deposition strategy with 10 s of dwelling time between two consequent paths (S2), the same trend was achieved, but the increasing trend was less steep because the heat accumulation was attenuated by the waiting time between depositions;
- In the one-way deposition strategy with 12.5 s of dwelling time, including 2.5 s for the return to the beginning of the single path (S3), the trend of the areas is approximately constant throughout the process. This is due to the combination of waiting times and constant deposition direction, which allow an effective diffusion of the accumulated heat;
- By analyzing the key points of each track it can be noticed that, regarding the melt pool size variation along with the single deposited layer, the S3 strategy is the most stable, showing an average variation of 1725 px, while the S1 strategy proved to be the most uneven with a maximum variation of more than 10,000 px;
- The average melt pool areas for the three deposition strategies were compared and the following outcomes were recorded—an increase of 1.781 mm^2^ for strategy S1, 0.682 mm^2^ for strategy S2, and 0.238 mm^2^ for strategy S3. These results have corroborated the considerations given above.

This study might be useful for improving the accuracy and quality of depositions performed with complex deposition strategies and geometries. Results showed that the region-based active contour algorithm is a fast and stable image processing implementation and a successful methodology for closed-loop monitoring in real-time applications.

## Figures and Tables

**Table 1:** Chemical composition of base metal (wt.%).

Material	Cr	Ni	Mn	Si	C	Fe
AISI 304 stainless steel	19.14	8.71	1.15	0.40	0.061	70.539

**Table 2:** Chemical composition of powder (wt.%).

Material	Cr	Ni	Mn	Si	Mo	C	Fe
AISI 316L stainless steel	16.62	11.48	2.0	0.7	2.64	0.025	66.535

**Table 3:** Particle size distribution (PSD) of the powder.

PSD	Particle Size (μm)
D10	19
D50	30
D90	46

**Table 4:** Constant process parameters.

Parameters	Unit	Notation	Value
Laser power	W	*P*	400
Translation speed	mm min^−1^	*v*	1000
Powder feed rate	g min^−1^	*Q*	10
Carrier gas flow rate	L min^−1^	*G*	10
Laser spot diameter	mm	*d*	1.5

**Table 5:** Comparison of the processing times and precision of the four selected image processing algorithms.

Parameters	Image	Region-Based Active Contour	Threshold Segmentation	Edge Detection: Canny Edge	Edge-Based Active Contour
Processing time (s)	I	0.8344	0.8544	1.2114	3.9432
	II	0.7746	0.8876	1.1152	3.7735
	III	0.8355	1.0280	1.2799	4.2391
	IV	0.8167	0.8570	1.1992	3.8467
Average processing time (s)	I–IV	0.8153	0.9067	1.2014	3.9506
Accuracy		Very good	Bad	Quite good	Acceptable

## Data Availability

The data presented in this study are available on request from the corresponding author.
